# Real-time snapping dynamics and nanoscale thickness profiling of salmon keratocyte tunneling nanotubes using partially coherent quantitative phase microscopy

**DOI:** 10.1038/s41598-026-46064-1

**Published:** 2026-04-17

**Authors:** Bilal M. Afzal, Marie K. Mikkelborg, Dhivya B. Thiyagarajan, Deanna L. Wolfson, Balpreet S. Ahluwalia, Roy A. Dalmo, Azeem Ahmad

**Affiliations:** 1 The Norwegian College of Fishery Science, UiT The Arctic University of Norway, 9037 Tromsø, Norway; 2 Department of Physics and Technology, UiT The Arctic University of Norway, 9037 Tromsø, Norway

**Keywords:** Biological techniques, Biophysics, Cell biology, Nanoscience and technology

## Abstract

Fish skin epithelial cells, known as keratocytes, are highly migratory and exhibit distinctive morphological features, including elongated membrane protrusions called tunneling nanotubes (TNTs). In other cell types, TNTs have been reported to facilitate the intercellular transfer of various cellular cargo. Beyond cargo transport, these structures are also implicated in mediating intercellular signaling. Owing to their extremely small heights, often as low as 100 nm or less, reliable characterization of their morphology and dynamics necessitates the use of a highly sensitive quantitative phase microscopy technique. In this study, a partially spatially coherent quantitative phase microscopy (QPM) system was utilized, providing extremely high spatial sensitivity to track changes in TNT height over time, particularly during the snapping process. A five-frame phase-shifting technique was employed to recover high-resolution phase maps of the TNTs. Observations indicate that TNT height typically ranges from 100 to 900 nm. Additionally, the average height-to-width ratio indicates that the TNTs exhibit a more flattened rather than cylindrical geometry. Further, during the imaging period, diverse behaviors were observed: some TNTs remained intact and maintained structural integrity for the entire duration of the recording, whereas others exhibited rupture. Beyond morphological characterization, this work may open new avenues for understanding TNT’s potential role in the transfer of intracellular cargo, paving the way for future investigations into cellular communication mechanisms.

**Supplementary Information:**

The online version contains supplementary material available at 10.1038/s41598-026-46064-1.

## Introduction

Atlantic salmon (*Salmo salar*) farming in Norway has progressed to 1.5 million metric tons in 2023^1^. Despite this progress, farmed salmon still face nearly 20% mortality on average, largely due to infectious pathogens, physical injuries, and various stressors^2^. While wound healing on the fish skin occurs following mucosal infection or injury, the cellular communications involved in this process remain poorly understood.

Studies on innate immunity in higher vertebrates suggest that cells communicate via ligands and receptors and through more direct cell-to-cell interactions^3^. Through direct communication, the cells may even exchange organelles such as mitochondria, which is beneficial when it comes to cellular rescue and regeneration^4^. One such direct path for cell communication is through special extended cytoskeletal protrusions, referred to as tunneling nanotubes (TNTs). These long membrane extensions may be a route of transfer of various small molecules, organelles, and even pathogens, including bacteria and viruses^5^. While TNT-like structures have previously been reported in different human immune cells, including B cells, T cells, and NK cells^6^. To the best of our knowledge, TNT formation has not previously been reported in primary fish skin epithelial cells, which is the specific biological context of this work.

In various human cells, such as kidney cells, monocytes, proximal tubular epithelial cells (RPTEC) from the kidney, and Jurkat T cells, TNTs connect cells over distances exceeding 100 μm^7–9^. Furthermore, it has been reported that in cultured rat pheochromocytoma PC12 cells (neuroblastic-like), these TNTs can have diameters ranging from 0.05 to 1.5 μm and lengths extending across multiple cell diameters^10^. TNTs have been documented in zebrafish, where TNT-like structures have been observed facilitating the transfer of Wnt proteins in embryos^11^. In related research, structures resembling TNTs were identified during a specific developmental stage of zebrafish, notably gastrulation. These structures facilitated the transport of proteins between distant cells^12^.

Live-cell label-free imaging provides a powerful platform for monitoring cellular dynamics in real time and examining interactions between cells, while avoiding artifacts that may arise from chemical fixation procedures^13^. Our methodology uses minimally phototoxic, label-free live cell imaging, which can be used to observe dynamic behavior and snapping events of the TNTs during cell migration. Given that TNTs are small, slender, and mechanically delicate structures, the use of a label-free optical microscopy technique capable of delivering high-contrast time-lapse imaging is critical. Alternative imaging modalities, particularly those requiring labeling or intense illumination, may perturb TNT structure and dynamics, potentially influencing the observed behavior.

Quantitative phase microscopy (QPM) is a non-invasive, non-contact, and label-free imaging technique highly compatible with live-cell studies. QPM generates contrast from the optical phase delay introduced by the specimen, i.e., from the optical path length (OPL) difference relative to the surrounding medium. This phase delay depends on both the refractive-index difference between the specimen and the immersion medium and the physical thickness of the specimen. Furthermore, it provides precise, quantitative measurements of various morphological and biophysical parameters, including volume, surface area, dry mass, mean thickness fluctuations, and height variations between cells^14,15^. Over the last two decades, variants of QPM have been developed, which can be classified into two main categories: common-path and non-common-path interferometric configurations^16,17^. A Linnik-based non-common-path interferometric QPM setup is used in this study. QPM encodes biological information, such as optical thickness (geometric thickness × refractive index), in the form of modulated intensity patterns known as interferograms.

One of the important parameters of a QPM system is its spatial phase sensitivity, which determines the minimum phase/height detection limit of the system^18–20^. Therefore, the spatial phase sensitivity of the system must be high to detect minuscule variations, e.g., down to 10 nm differences in height, in biological specimens, as in the case of TNTs due to their sub-micrometer height. Here, the choice of the light source plays a crucial role in determining the phase sensitivity of the system^19^. With a fully coherent laser light source, it is quite easy to form interference fringes in a QPM system, but at the cost of significant speckle noise and parasitic fringe patterns, resulting in reduced spatial phase sensitivity. The highest phase sensitivity in a QPM system can be achieved by implementing low-coherence light sources such as halogen lamps and LEDs^18,20^. However, with this type of light source, obtaining interference fringes is challenging due to their short temporal coherence length (a few microns). The interference fringes are observed only when the optical path difference between the object and reference arms lies within the coherence length of the source used in the interferometer. These issues are addressed by implementing a pseudo-thermal light source (PTLS), which has low spatial coherence and high temporal coherence^21–24^. PTLS, also called dynamic speckle illumination, is generated by passing the laser beam through a rotating diffuser (RD). This configuration produces a light source whose statistical properties closely resemble those of broadband incoherent sources such as halogen or white light. In particular, the intensity statistics of PTLS follow thermal (Gaussian) statistics rather than coherent laser statistics, a behavior that has been well characterized in the literature^25^. With PTLS, high spatial phase sensitivity comparable to that of low-coherence light sources can be achieved while also maintaining the ease of forming interference fringes. In addition, PTLS enables high space-time bandwidth product imaging in the QPM system^19,26^. Owing to these advantages, a PTLS-based QPM configuration was adopted for the present study to ensure highly sensitive phase imaging of delicate cellular structures.

In principle, the temporal resolution of our system is limited by the camera speed when operated in single-shot mode, which requires the formation of high–fringe-density interferograms for Fourier-transform–based phase recovery^27^. In the present work, however, we employed temporal phase-shifting reconstruction algorithms, which provide diffraction-limited spatial resolution and superior phase reconstruction quality at the expense of reduced temporal speed. Because the biological dynamics of fish cells do not require extremely high acquisition rates, we prioritized spatial resolution and measurement fidelity over temporal speed.

In this study, because the individual phase steps are neither fixed nor known a priori, a principal component analysis (PCA) based phase-shifting algorithm is employed. This method enables accurate phase recovery without requiring fixed or calibrated phase steps and is robust to random and unknown phase shifts. Owing to its underlying singular value decomposition (SVD) framework, the PCA-based method separates background illumination from phase modulation, which improves reconstruction stability. In addition, the approach is relatively insensitive to temporal instabilities in the optical system, including vibrations and turbulence, making it suitable for time-lapse measurements. Importantly, the principal eigenvalues obtained from the PCA provide intrinsic phase-quality indicators, allowing blind assessment of reconstruction reliability. These properties make PCA-based quantitative phase reconstruction well suited for label-free, time-lapse imaging of dynamic cellular structures^28–32^. Thus, the combination of pseudo-thermal illumination, phase-shifting interferometry, and PCA-based reconstruction enables stable interferometric measurements with high phase sensitivity while allowing long-duration, label-free live-cell imaging with minimal phototoxicity. These advantages are particularly relevant for TNT measurements, where fragile nanoscale structures must be monitored over extended time periods.

In this study, analysis of the average height-to-width ratio reveals that TNTs exhibit a predominantly flattened rather than ideal cylindrical geometry, suggesting deviations from circular symmetry that may reflect underlying mechanical or membrane-related constraints. In addition, time-lapse imaging of TNTs revealed heterogeneous structural dynamics. During the observation period, some TNTs are unbroken and maintained structural integrity throughout the entire recording, whereas others underwent rupture. These findings emphasize the dynamic and, in some cases, transient nature of TNTs. Thus, the present work provides a quantitative framework for investigating TNT structure and dynamics in live fish cells. Such measurements may facilitate future studies aimed at elucidating the potential role of TNTs in intracellular cargo transfer and intercellular communication, thereby contributing to a deeper understanding of cell–cell interaction mechanisms.

## Materials and methods

### Sample preparation

Post-smolt Atlantic salmon (500–2 kg, mixed sexes) were obtained from Tromsø Aquaculture Research Station, Kårvika, Norway. The fish were kept in seawater (33–34 ppt salinity, 3–9 °C, natural light) and received commercial diets (Skretting Spirit Trout 4.5, Celero 4.5). Ten fish were sampled during different periods of the year, all unvaccinated. After sanitizing equipment with Virkon, the fish were euthanized with a cranial blow and transported to the Norwegian College of Fishery Science, UiT, Tromsø. Scales were collected from one to two fish per experiment. The procedures followed Norwegian regulations for animal experimentation (Forskrift 2015-06-18-761) and EU Directive 2010/63/EU, allowing the use of unregulated post-mortem samples without FOTS applications.

For imaging, salmon keratocyte samples were prepared on silicon wafers. A custom-made Polydimethylsiloxane (PDMS) chamber (12 mm × 12 mm, 150 μm thick) was applied to the wafer surface. Fish scales were collected from various skin dorsoventral areas using sterile tweezers and seeded onto the substrate with the interior surface in contact with the substrate. Scales adhered to the surface within approximately six minutes, after which they were exposed to 200 µL of Hank’s Balanced Salt Solution (HBSS) (VWR, 21-023-CM) mixed with antibiotics, 100 µg/ml streptomycin, 100IU/ml penicillin (Sigma-P0781), and 1 µg/ml of Amphotericin B solution (Sigma-A2942). Samples were incubated at 12 °C, cell avalanches were monitored for 2–4 days, and imaging was performed. Although the typical rearing temperature for Atlantic salmon is 8–10 °C, primary cells can be successfully cultured across a broader temperature range (2–20 °C). Based on experimental optimization, a culture temperature of 12 °C was selected for all experiments, as lower temperatures markedly reduce cell migration from the scales to the substrate, whereas 12 °C is optimal for obtaining migrating cells from the fish scales.

### PCA-based asynchronous phase-shifting interferometry

In phase-shifting interferometry, an intensity sequence of $\:N$ interferograms can be written as^33^:

1 $$\:\begin{array}{cccc}&\:{I}_{n}(x,y)=a(x,y)+b(x,y)\mathrm{c}\mathrm{o}\mathrm{s}\left[{\Phi\:}\right(x,y)+{\delta\:}_{n}]\end{array}$$

where $\:a(x,y)$ denotes the background term, $\:b(x,y)$ the fringe modulation, $\:{\Phi\:}(x,y)$ the object phase, and $\:{\delta\:}_{n}$the unknown phase shifts.

Using the trigonometric identity for cosine addition,

2 $$\:\begin{array}{cccc}&\:{I}_{n}=a+b\left[\mathrm{c}\mathrm{o}\mathrm{s}\left({\Phi\:}\right)\mathrm{c}\mathrm{o}\mathrm{s}\left({\delta\:}_{n}\right)-\mathrm{s}\mathrm{i}\mathrm{n}\left({\Phi\:}\right)\mathrm{s}\mathrm{i}\mathrm{n}\left({\delta\:}_{n}\right)\right]\end{array}$$

After subtracting the background component $\:a$, the interferogram $\:{\stackrel{\sim}{I}}_{n}$ can be written as a linear combination of two quadrature fields:

3 $$\mathop I\limits^{\sim } _{n} = \alpha \:_{n} I_{c} + \beta \:_{n} I_{s}$$

with

$$\begin{aligned} & \:{\alpha\:}_{n}=\mathrm{c}\mathrm{o}\mathrm{s}\left({\delta\:}_{n}\right),\:\:{\beta\:}_{n}=\mathrm{s}\mathrm{i}\mathrm{n}\left({\delta\:}_{n}\right), \\ & \:{I}_{c}=b\mathrm{c}\mathrm{o}\mathrm{s}\left({\Phi\:}\right),\:\:{I}_{s}=b\mathrm{s}\mathrm{i}\mathrm{n}\left({\Phi\:}\right). \end{aligned}$$

Thus, each interferogram can be decomposed into two uncorrelated (i.e., orthogonal) quadrature components $\:{I}_{c}$ and $\:{I}_{s}$ and satisfy the following expression:

4 $$\mathop I\limits^{\sim } _{n} = \alpha \:_{n} I_{c} + \beta \:_{n} I_{s} \approx 0$$

where Nx × Ny is the image size.

Let each interferogram be vectorized into a column vector $\:{x}_{n}\in\:{\mathbb{R}}^{{N}_{x}{N}_{y}}$. The complete dataset of N interferograms of size Nx × Ny is arranged as

5 $$X = [x_{1} ,x_{2} , \ldots \:,x_{N} ]^{T}$$

After mean subtraction,

$$\:\grave{X}=X-{m}_{X},$$

where $\:{m}_{X}$ contains the column-wise mean values, the covariance matrix is computed as

6 $$C=\grave{X}{\grave{X}}^{T}$$

Since $\:C$ is real and symmetric, it possesses a complete set of real eigenvalues along with mutually orthonormal eigenvectors.

7 $$C={A}^{T}DA$$

where $\:D$ is diagonal (eigenvalues) and $\:A$ contains orthonormal eigenvectors. The principal components are obtained through the Hotelling transform^34^:

8 $$Y=A \grave{X}$$

Because the interferometric signal is intrinsically two-dimensional (cosine and sine quadrature), the first two principal components (corresponding to the largest eigenvalues) approximate $\:{I}_{c}$ and $\:{I}_{s}$.

The phase map is finally reconstructed as

9 $${\Phi\:}(x,y)={\mathrm{t}\mathrm{a}\mathrm{n}}^{-1}\left(\frac{{I}_{s}(x,y)}{{I}_{c}(x,y)}\right)$$

The global phase sign remains ambiguous due to arbitrary assignment of cosine and sine components to the principal directions.

### Experimental setup

**Fig. 1:**
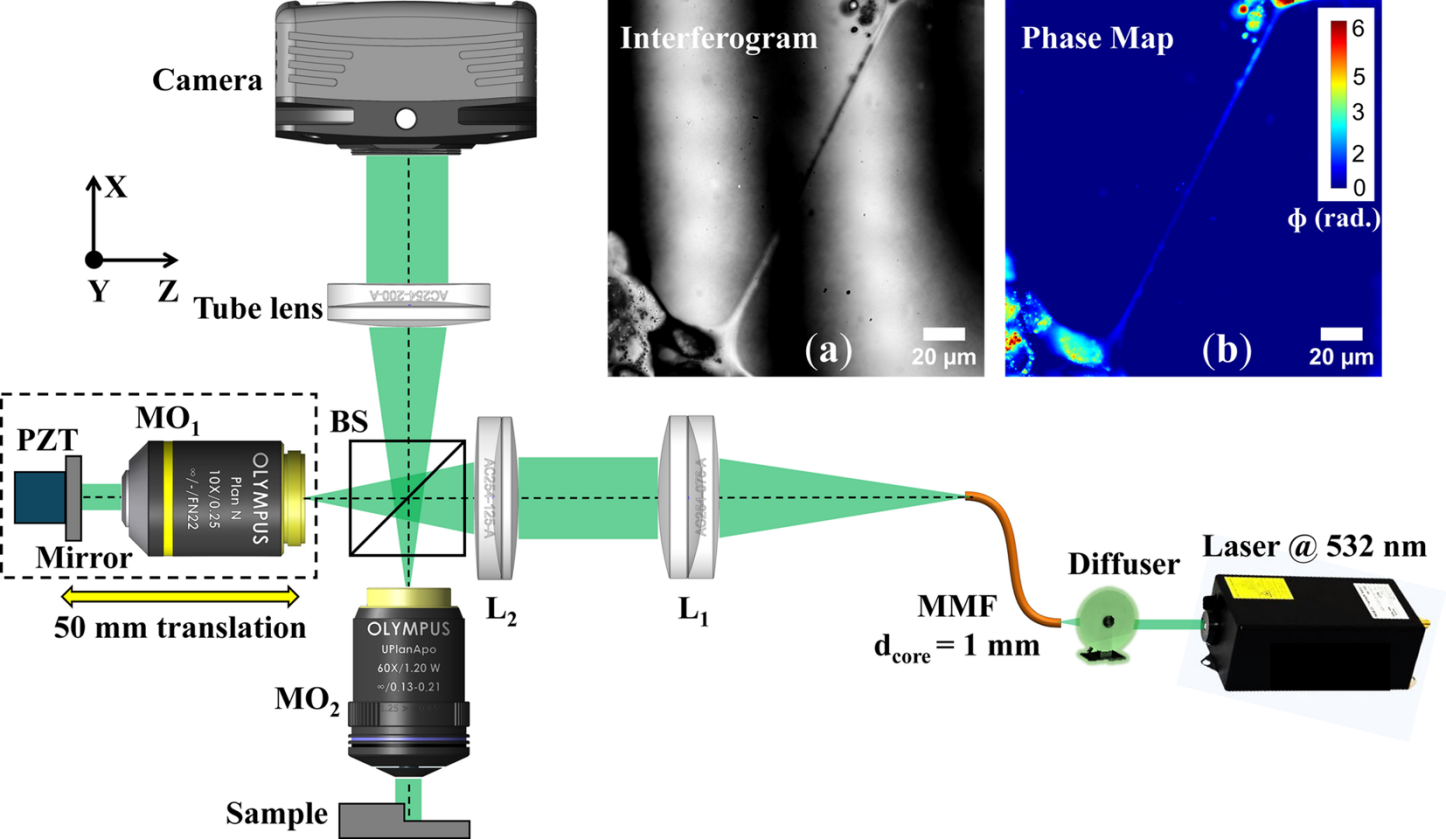
Schematic of the Linnik interferometry-based quantitative phase microscope with a pseudo thermal light source (PTLS-QPM). MMF, multimode fiber; L1 and L2, achromatic doublet lenses; BS, beam splitter; MO, microscope objective; PZT, piezoelectric transducer. Panel (**a**) shows one of the phase-shifted interferograms captured by the camera, and Panel (**b**) displays the recovered phase map. The color bar indicates the phase values in radians.

Imaging data were acquired using a custom-built QPM system to thoroughly investigate TNTs in keratocytes. Figure 1 illustrates the Linnik interferometer-based QPM setup. The details of the setup can be found in Refs^19,35^. A pseudo-thermal light source (PTLS) was generated by directing a 532 nm laser beam (Cobolt Flamenco laser) onto a rotating diffuser, followed by a multimodal fiber (MMF), as shown in Fig. 1. The output of MMF was collimated through a lens (L1) and focused by a second lens (L2) at the back focal plane of the sample arm microscope objective lens (60×/1.2NA water immersion, model # UPlanSApo, Olympus). The beam splitter divides the input beam into two, one directed toward the sample arm, known as the sample beam, and the other toward the reference arm, known as the reference beam. The sample beam interacts with the sample, and its information is collected by the same objective lens. The reference beam is passed through the microscope objective lens 10×/0.25NA and is reflected from the reference mirror. Both light beams are recombined at the beam splitter plane to form interference fringes, which are then projected onto a camera using a tube lens. A piezoelectric stage is used in the reference arm to introduce phase stepping between consecutive frames. The acquisition of five temporal phase-shifted frames is performed using a Hamamatsu CMOS camera (C11440-42U) with an effective number of pixels of 2048 (H) × 2048 (V) and a pixel size of 6.5 μm. The acquisition time required to capture five phase-shifted frames is approximately 600 ms at full frame and is performed using Micro-Manager. The sample is mounted on a motorized XYZ translation stage to select a region of interest. Previously, the developed system has demonstrated its effectiveness in studying the association of microplastics with salmon keratocytes^36^.

To capture the dynamic behavior of TNTs, five temporal phase-shifted interferograms of fish keratocytes forming TNTs were recorded and subsequently post-processed to reconstruct corresponding phase maps using the principal component analysis (PCA) algorithm^29,30,33^. The recovered phase is then numerically focused to minimize errors in the calculation of morphological parameters related to TNTs.

### Geometrical height calculation

To quantify TNT height, a line profile with a width of 100 pixels was drawn perpendicular to the TNT length. In total, the height variations of 12 TNTs (6 broken and 6 unbroken) were measured. Each TNT trajectory comprises hundreds of time points, providing robust estimates of temporal behavior at the level of individual structures. Phase values were extracted along this line for each frame to monitor changes in the phase map over time. Geometrical height (GH) was then calculated by applying the following equation:

10 $$\:H\left(x,y\right)=\frac{\lambda\:}{4\pi\:\left({n}_{s}-{n}_{m}\right)}{\Phi\:}\left(x,y\right)$$

where $\:{\Phi\:}\left(x,y\right)$ represents the measured phase map of the specimen, λ is the wavelength of the illumination light, $\:{n}_{s}$ is the refractive index of the sample, and $\:{n}_{m}$ is the refractive index of the surrounding medium. For the calculation of the GH of the TNTs, $\:{n}_{s}$=1.38 and $\:{n}_{m}$=1.33 were assumed. The quantitative phase measurements were interpreted using a standard effective RI approximation ($\:{n}_{s}$=1.38), which is commonly employed in quantitative phase microscopy studies of cellular structures. Importantly, this approximation does not affect the observed temporal trends in TNT thickness; it only influences the absolute numerical values of the estimated height.

### Spatial and temporal phase sensitivity analysis

**Fig. 2:**
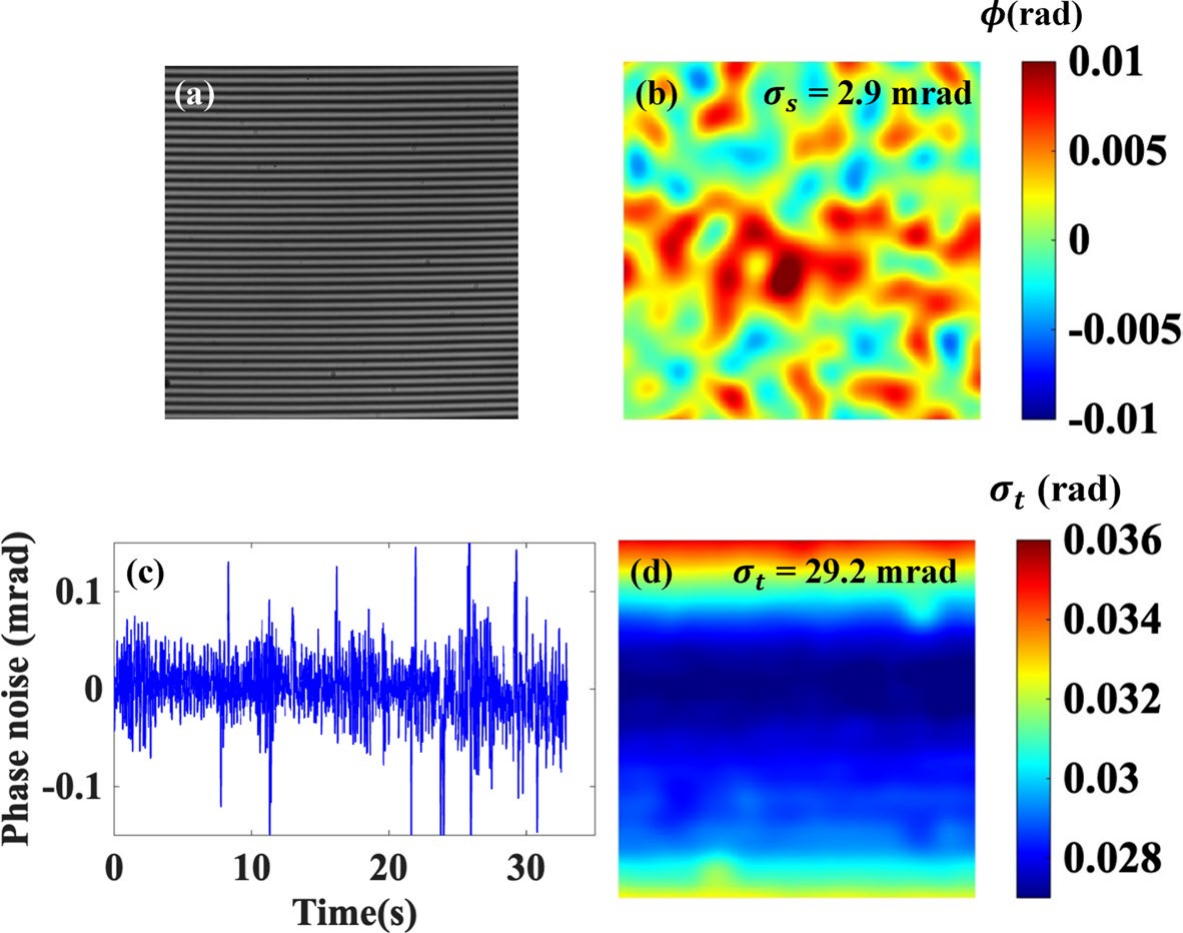
Spatial and temporal phase sensitivity analysis of the QPM system. (**a**) Representative interferogram extracted from a 35 s interferometric movie recorded at 33 frames per second (see Supplementary Video V1). (**b**) Corresponding reconstructed quantitative phase map, from which a spatial phase sensitivity of 2.9 mrad was obtained, corresponding to a height variation of 2.4 nm. (**c**) Temporal phase fluctuation at a representative pixel location (x, y) = (10, 10) after removal of global phase drift. (**d**) Spatial distribution of the standard deviation of temporal phase fluctuations over the entire FOV. The mean temporal phase sensitivity was measured to be 29.2 mrad, corresponding to a height variation of 24.7 nm.

To evaluate the detection performance of the QPM system, both spatial and temporal phase sensitivities were quantified. An interferometric movie with a duration of 60 s was recorded at a frame rate of 33 frames per second (see Supplementary Video V1). A representative interferogram and its corresponding reconstructed phase map are shown in Fig. 2a,b, respectively. The spatial phase sensitivity was determined from the standard deviation of the phase distribution in a background region of the reconstructed phase map. The measured spatial phase sensitivity ‘$\:{\sigma\:}_{s}$’ of the system was 2.9 mrad. This phase sensitivity corresponds to a geometrical height measurement accuracy of 2.4 nm, assuming refractive indices of 1.38 for the cells and 1.33 for the surrounding medium at an illumination wavelength of 532 nm.

To assess temporal phase stability, all interferometric frames of the recorded movie were processed to retrieve the corresponding phase maps. The temporal phase fluctuation ‘$\:{\sigma\:}_{t}$’ at each pixel was then calculated over time. To remove errors caused by global phase drift, the temporal phase profile averaged over the entire field of view (FOV) and then subtracted from the temporal phase profiles at each pixel location. An example of the temporal phase fluctuation at a representative pixel location (x, y) = (10, 10) is shown in Fig. 2c. The spatial distribution of temporal phase fluctuations over the entire FOV is presented in Fig. 2d. The mean value of the standard deviation of the temporal phase fluctuation across the FOV was measured to be 29.2 mrad, which corresponds to a height variation of 24.7 nm.

It is important to note that this level of temporal height fluctuation does not introduce significant error in the quantification of TNT height. In practice, the effect of temporal noise is further minimized by subtracting the local background phase in the vicinity of the TNT region prior to height measurement. This background correction procedure is systematically applied to all phase movies used for TNT height quantification, thereby ensuring reliable and accurate measurements across all cells.

### Background phase estimation and subtraction

The background removal procedure was performed using a rolling-disk algorithm^37^. The diameter of the disk was set to 30 pixels, which was empirically chosen to ensure that structural information related to the sample was preserved and not removed during background subtraction. This disk size was found to be sufficient for accurate background estimation in the case of TNTs, whose characteristic dimensions are much smaller than the selected disk diameter. Based on our experience, when applying this method to larger cellular structures, the disk diameter must be increased to a value greater than half of the average cell size to avoid attenuation of biologically relevant features. This adaptive selection of disk diameter ensures effective suppression of low-frequency background variations while maintaining the integrity of fine sample-related phase information, thereby enabling reliable quantitative height measurements.

## Results and discussion

### Quantitative phase imaging of salmon keratocyte TNTs

Tunneling nanotubes (TNTs) are typically described as long, thin membranous structures that physically interconnect cells over extended distances, contain continuous F-actin filaments, and remain suspended above the substrate rather than adhering to it^7^. In the present study, fluorescence experiments were performed independently to confirm TNT identity and validate their actin-based structure. However, the primary focus of this manuscript is the implementation of a label-free QPM approach and its capability to resolve the nanoscale morphology and temporal dynamics of TNTs.

While fluorescence-based labeling is widely used for TNT identification, it may introduce several limitations, particularly in long-term live-cell imaging. Fluorophore labeling can alter cellular physiology, affect membrane or cytoskeletal dynamics, and potentially influence the mechanical stability of delicate structures such as TNTs. In addition, photobleaching, phototoxicity, and background fluorescence may compromise prolonged observation, especially when monitoring subtle nanoscale height fluctuations or snapping events. Given the fragile and highly dynamic nature of TNTs, minimizing external perturbations was therefore essential for reliable quantitative analysis. For these reasons, fluorescence imaging was not systematically performed for all TNTs analyzed in this work. Instead, label-free QPM was employed to ensure minimally invasive, time-resolved measurements of TNT geometry and dynamics.

Experiments were conducted on salmon keratocytes using a PTLS-QPM system to quantify the GH of TNTs formed between cells. We confirm that collecting fish and removing scales does not require specific ethical approval under the Norwegian Regulations for the Use of Animals in Research (https://lovdata.no/dokument/SF/forskrift/2015-06-18-761#KAPITTEL_10). The procedure is also fully aligned with EU Directive 2010/63/EU (https://eurlex.europa.eu/legal-content/EN/TXT/?uri=CELEX:32010L0063). All experimental steps adhered to the applicable guidelines and regulatory frameworks, and the reporting follows the ARRIVE recommendations (https://arriveguidelines.org). According to our protocol, this work can be conducted without a separate FOTS (Mattilsynet) approval.

The system’s high contrast and nanometric optical path sensitivity enable detailed mapping of TNT morphology and its spatial connectivity between adjacent cells. TNTs appear as thin, elongated connections bridging keratocytes, with measurable phase shifts that reflect variations in height or subcellular mass density along their length. Such label-free visualizations are essential for investigating TNT dynamics and their functional roles in live cell interactions. Videos of up to 1000 timepoints with 600 ms time interval (Supplementary videos V2–V7), each with five temporal phase-shifted interferograms, were recorded and post-processed as reported in Materials and Methods.

**Fig. 3:**
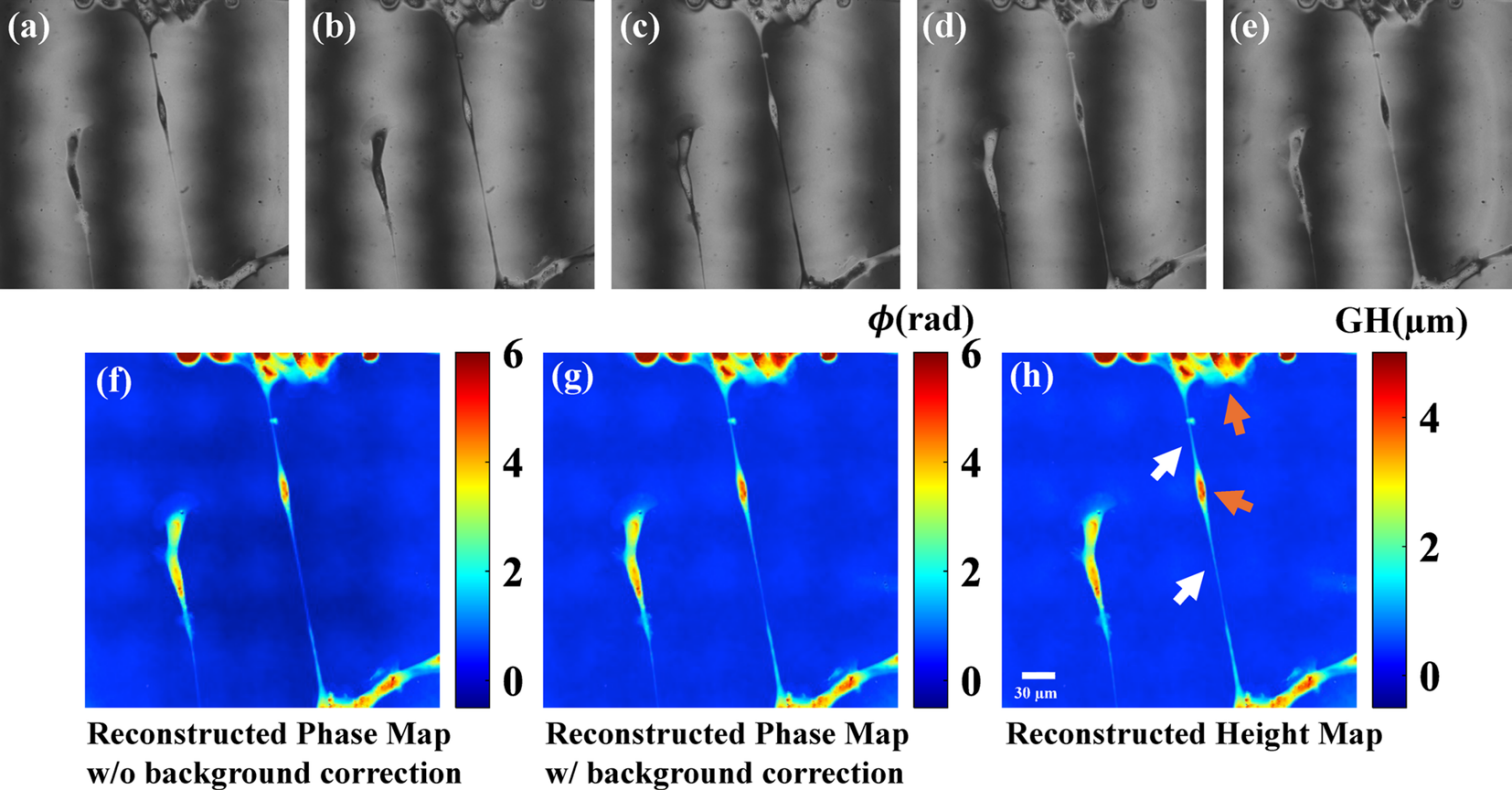
The high resolution of the PTLS-QPM system allows for the precise visualization of fine connections between cells. (**a**–**e**) 5 phase-shifted interferometric images of a salmon TNT connecting two keratocytes, captured using the PTLS-QPM system. (**f**) Reconstructed phase map from the interferometric image without global background correction. (**g**) The reconstructed phase map with global background correction. The color bar indicates the phase in radians ($\:\varphi\:)$. (**h**) This map displays the estimated GH of the TNT. The color bar indicates the GH values in nm. White arrows point to the TNT connecting cells, and orange arrows show cell bodies. The corresponding movie can be found in the Supplementary Video V5. A 60× / 1.2 NA water immersion objective is utilized for imaging.

An example of a 5-phase-shifted interferogram is shown in Fig. 3a,e. The corresponding reconstructed phase maps without global background correction and with global background correction are presented in Fig. 3f,g, respectively. The respective GH map in µm is shown in Fig. 3h. These images show the presence of TNTs connecting distant keratocytes, with the height of the highlighted TNT around 100 nm. Notably, the height of TNTs varies between cells (see Table 1), potentially reflecting differences in the nature or size of the cargo being transported. Cells may form TNTs with distinct height profiles to facilitate the transfer of specific organelles or molecular complexes, suggesting functional adaptation in their structural morphology^38^.

**Table 1:** This table represents TNT’s mean geometrical height and standard deviation calculated from 6 different time lapse movies.

Movies	Broken TNTs GH (nm)		Unbroken TNTs GH (nm)	
	Mean	STD (±)	Mean	STD (±)
1	122	13	239	15
2	578	83	288	23
3	338	2	912	23
4	123	15	107	24
5	256	18	389	28
6	165	13	152	6

### Height of broken vs. unbroken TNTs

**Fig. 4:**
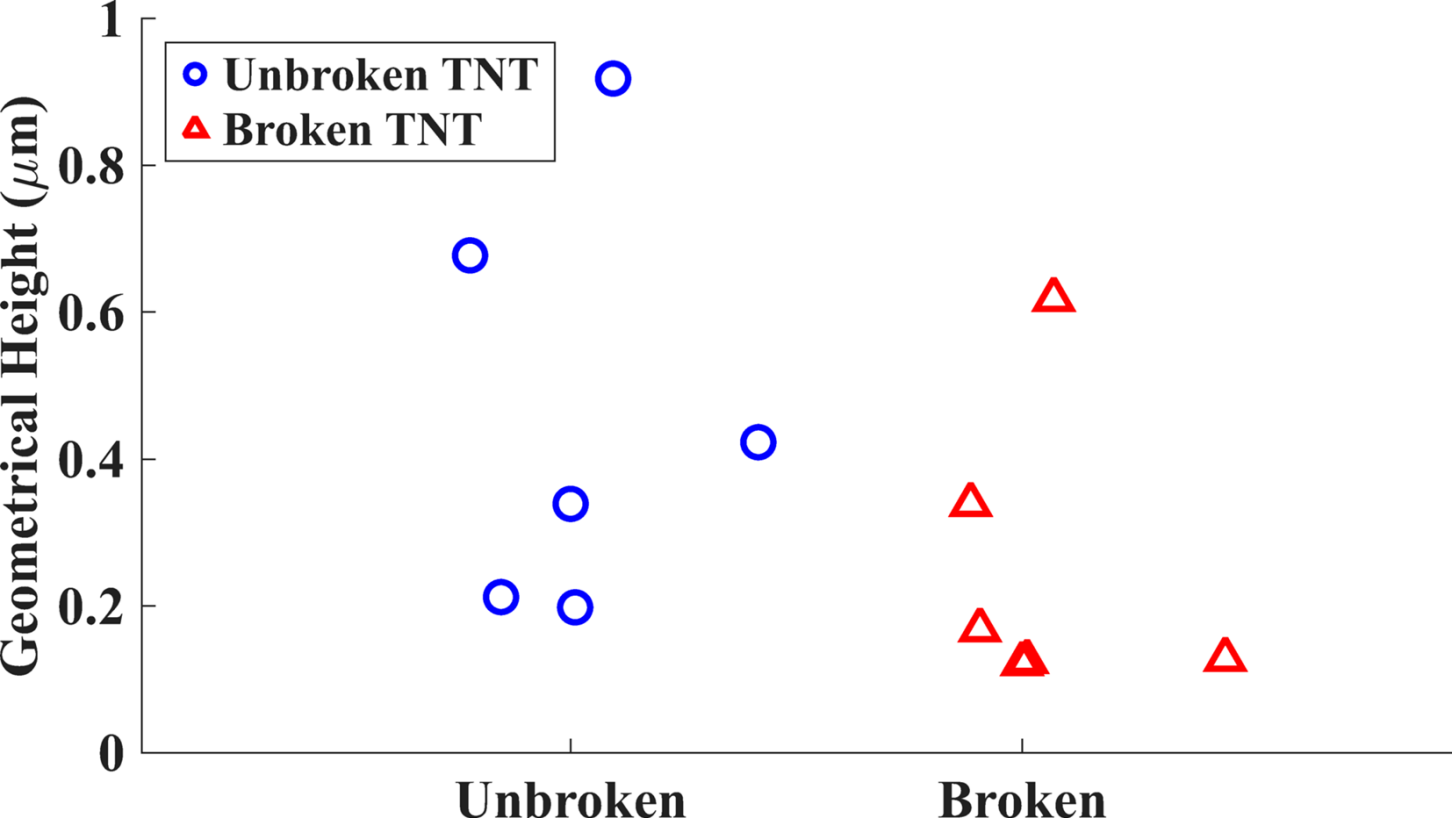
Comparative analysis of average heights: Unbroken vs. Broken TNTs. This plot illustrates the average heights of six unbroken TNTs against six TNTs prior to breaking, showing a higher range of height for the unbroken TNTs. The maximum value of the unbroken TNTs reaches up to 900 nm, suggesting potential structural resilience in TNTs with greater thickness. This analysis indicates that thicker TNTs may be more resistant to environmental stress.

During the observation period, distinct structural behaviors were noted: some TNTs remained intact and preserved their structural integrity throughout the entire imaging sequence, whereas others underwent rupture. In this section, the height profiles of broken and unbroken TNTs were quantitatively analyzed. Figure 4 compares the GH profiles of six unbroken TNTs with six TNTs that underwent rupture. Each TNT trajectory contains hundreds of time points, allowing reliable characterization of temporal height trends with primary focus to measure the variation in TNT height immediately preceding the snapping event.

Unbroken and broken TNTs exhibited overall GH within similar ranges, with maximum heights of approximately 900 nm and 700 nm, respectively. Across all measurements, TNT heights ranged from roughly 100 to 900 nm. Quantitative height statistics for individual TNTs are summarized in Table 1. For rupturing TNTs, the mean GH and variability were calculated from eight consecutive frames immediately preceding rupture, while the final two frames were excluded to avoid measurement artifacts associated with snapping. For unbroken TNTs, the mean GH ± SD was calculated from ten consecutive frames. In total, twelve independently tracked TNTs (six broken and six unbroken) were analyzed, with summary statistics reported for each individual TNT. The mean TNT height, calculated from six broken TNT movies prior to snapping, was 263 ± 24 nm (mean ± SD, *n* = 6 broken TNTs). Similarly, the mean GH of TNTs, calculated from six unbroken TNT movies, was 348 nm ± 293 nm (mean ± SD, *n* = 6 unbroken TNTs). It is important to emphasize that these measurements are descriptive and are not intended to establish population-level statistical significance; comprehensive population-based analysis may be conducted in future studies.

Note that QPM was performed at extremely low illumination levels (fluence of ~ 3.1 × 10^−5^ J/cm^2^ for the total acquisition time of 600 ms), corresponding to energy fluences several orders of magnitude below reported photothermal damage thresholds in live cells. For example, photothermal injury has been observed only at fluences in the range of ~ 0.5–90 J cm^−2^ under laser excitation in various cell types, whereas the illumination levels used here remain far below these thresholds^39^. Therefore, under such a low illumination level, illumination-induced rupture of TNTs is not expected.

These results suggest a strong correlation between TNT heights and structural resilience. Thicker TNTs may possess increased mechanical stability, potentially due to enhanced cytoskeletal reinforcement or membrane composition, making them more resistant to stress-induced rupture. In human monocyte-derived macrophages, researchers have observed two distinct types of tunneling nanotubules (TNTs): thinner TNTs, approximately 300 nm in diameter, composed solely of F-actin, and thicker TNTs, around 800 nm, which contain both F-actin and microtubules. The thicker TNTs are capable of transporting organelles such as mitochondria, endosomes, and lysosomes^38^. Thinner TNTs in fish may be involved in the transfer of smaller organelles, calcium ions (Ca^2+^), and the propagation of long-distance electrical signals^6,7^. This trend highlights the significance of geometrical and mechanical parameters in determining TNT integrity and lifespan. The formation and breaking of TNTs are a continuous process as the epithelial cells move around. The analysis revealed that some TNTs exhibited progressive height variations over time and ultimately snapped. These findings highlight the dynamic and mechanically sensitive nature of TNTs. Further investigations are required to identify the factors governing TNT stability and rupture and to better understand their potential roles in fish immunological processes.

For thin structures such as TNTs, optical path length (OPL)-based phase measurements provide a reliable estimate of their effective thickness within the range observed in this study (~ 100–900 nm), without requiring axial image stacking. Under partially coherent illumination, the effective depth of focus (DOF) of the interferometric imaging system is governed by the spatial coherence of the source and the numerical aperture of the imaging objective lens. For the experimental parameters used here (NA = 1.2, λ = 532 nm, and a partially coherent fiber-delivered source), the effective DOF is approximately ~ 1.3 μm, calculated using the formula provided in Ref^40^. Since the TNT heights measured in this work (≈ 100–900 nm) remain well within this axial range, the imaging objective can accurately capture the object wavefront without the need for axial scanning. Consequently, the phase-derived height represents the transverse line profile of the entire TNT thickness rather than a partial section, and the reported phase measurements are not limited by depth-of-focus constraints of the imaging system.

Further, phase wrapping errors are also not expected in the present TNT height measurements. Phase wrapping occurs when the measured phase exceeds 2π, which is primarily determined by the optical path difference of the specimen rather than the DOF of the imaging system. In practice, a limited DOF may indirectly introduce phase errors when structures extend beyond the focal region, as defocus can introduce additional phase curvature and increase the apparent optical path difference. However, for the TNT structures investigated here (≈ 100–900 nm), the corresponding phase shifts are approximately 0.04π–0.34π, which remain well below the 2π wrapping threshold. Furthermore, because the effective DOF of the system (~ 1.3 μm) exceeds the TNT thickness, these structures remain within the focal region, thereby minimizing defocus-induced phase artifacts.

Furthermore, during live-cell imaging, no hardware autofocus system was employed. Instead, a numerical defocus-correction approach based on angular spectrum propagation was applied, enabling computational refocusing of the reconstructed complex field retrieved by QPM. This procedure compensates for small defocus-related deviations in the recovered phase and height maps during time-lapse imaging, ensuring stable and reliable TNT height measurements throughout the observation period.

### Height measurement of broken TNTs as a function of time

**Fig. 5:**
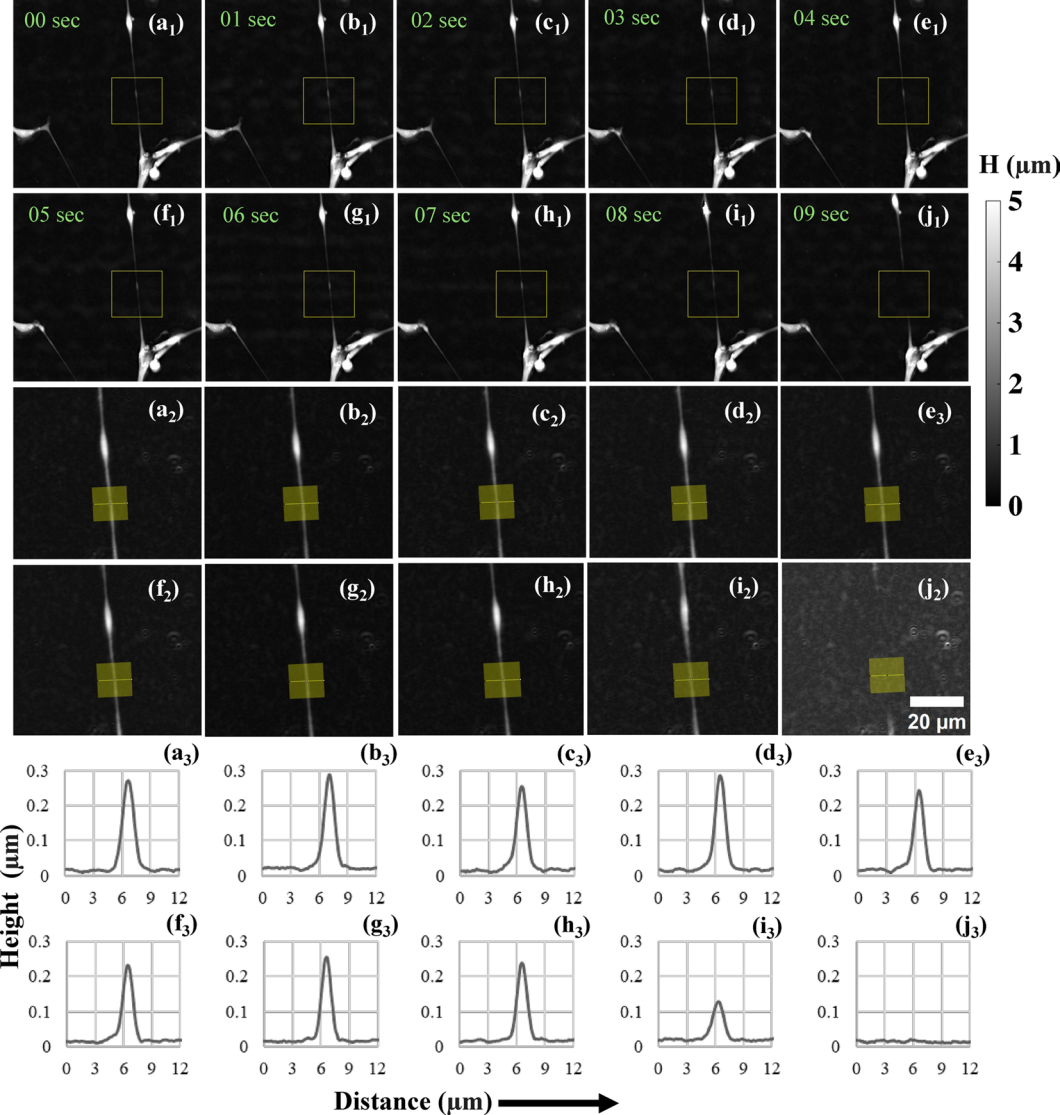
Changes over time in the height of 10 frames from a representative movie, including one frame captured immediately after a snapping event. (**a**_**1**_–**j**_**1**_) Reconstructed phase map images, with a yellow box highlighting the specific ROI measured. (**a**_**2**_–**j**_**2**_) show the measured area (yellow box) within the specific ROI, with measurements taken across 100 pixels perpendicular to the length to determine the GH. (**a**_**3**_–**j**_**3**_) Line profiles measured from the ROIs illustrate the variations in thickness over the 10 frames. Notably, the dynamic behavior of TNT thickness varies among different TNTs, and this figure contains only one example.

The temporal evolution of TNT height was analyzed using a sequence of ten frames from a representative movie: nine frames preceding a TNT rupture event and one frame captured immediately after the break. Figure 5a_1_–j_1_ presents the reconstructed phase maps for each time point, where the yellow box indicates the specific region of interest (ROI) in which the TNT breakage occurs. This ROI was applied across all frames using the ROI Manager in ImageJ, enabling accurate cropping and analysis (see frames in Fig. 5a_2_–j_2_.

The evolution of TNT height is visualized through line profiles from frames (Fig. 5a_3_–j_3_), revealing a clear trend for this TNT: the height decreases from approximately 0.27 μm in the early frames (Fig. 5a_3_) to below 0.1 μm (Fig. 5i_3_), just prior to rupture.

The dynamic behavior of TNT height varied across different recordings. In some movies, TNT height showed noticeable fluctuations over time (as illustrated in Fig. 4), while others exhibited consistent height throughout the sequence (see example in Supplementary Fig. S1). All TNTs included in this study were analyzed following the same procedure described above. The representative movies are provided in Supplementary Videos (V2–V7). Note that the phase movies are shown without background subtraction to preserve the raw reconstructed phase data; applying rolling-ball subtraction would reduce low-frequency fluctuations and flickering without affecting the underlying structural information.

### Quantitative temporal analysis of broken TNT’s height and height-to-width ratio (H/W)

**Fig. 6:**
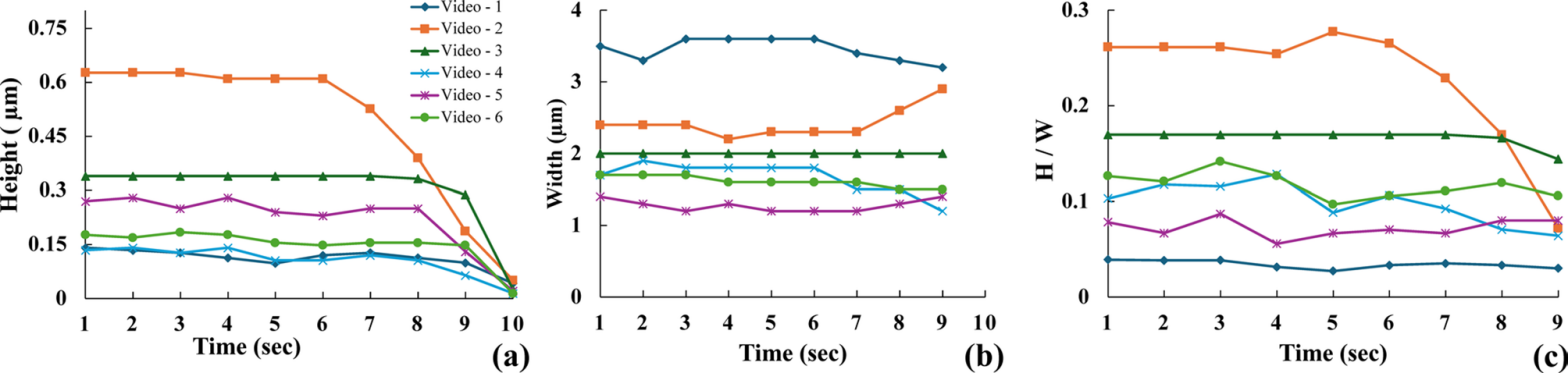
The higher phase sensitivity of the PTLS-QPM setup is used to quantify the GH variation and shape determination in TNTs. (**a**) This graph presents the average height variation across six different TNTs just prior to rupturing, each analyzed for 10 frames. The last frame of each sequence shows the height after snapping, which typically approaches zero. (**b**) This graph displays the width of the TNTs across the sequences. (**c**) displays the ratio of H/W of TNTs. This ratio suggests that the TNTs have a more flattened shape rather than a round.

The average height variation of broken TNTs over time is shown in Fig. 6a, based on measurements from different TNTs across ten frames of six representative movies, all of which included TNT snapping events. Complete video datasets are available in the Supplementary Information. The apparent red–blue “phase jumps” observed in certain regions of the cells in Supplementary Videos are not indicative of abrupt structural changes. These discontinuities arise from phase wrapping effects inherent to interferometric phase measurements. In optically thicker regions, particularly near the nuclear area, the accumulated optical path delay approaches or exceeds the principal phase interval (−π to π), resulting in wrapped phase discontinuities that appear as sudden color transitions. In regions where the signal-to-noise ratio (SNR) is relatively low, the phase unwrapping algorithm becomes less reliable due to increased sensitivity to noise and local phase gradients. Consequently, the displayed phase maps retain localized wrapping discontinuities in those areas. Importantly, these effects are confined to thicker cellular regions with reduced SNR. They are not observed along the analyzed TNT structures, where the phase gradients are smaller, and the SNR is sufficiently high to ensure stable and continuous phase reconstruction.

The results from Fig. 6a suggest that snapping of TNTs is mostly a sudden phenomenon and not a slow gradual decrease of height. The high sensitivity of the PTLS-QPM system allowed for the detection of the variation in TNT height leading to rupture. After the breaking event, the measured height of the TNTs nearly dropped to the height sensitivity limit, which is around 2.4 nm. Furthermore, on average, the GH of TNTs is around 305 nm ± 22, but in some cases, TNTs displayed heights exceeding the typical range, to around 900 nm (Table 1), which may indicate the presence of larger transported materials or specialized structural adaptations, as it happens in human cell TNTs^41^. The widths of the six TNTs presented in Fig. 6b demonstrate minor fluctuations, indicating that the width remains relatively consistent across the samples over time.

Further, structural parameters such as the height-to-width (H/W) ratio may provide valuable insight into the functional adaptations and biomechanical integrity of TNTs during dynamic cellular processes. In this analysis, the H/W ratio was quantitatively measured and tracked over 10 s for six individual movies of TNTs. As shown in Fig. 6c, each TNT exhibits dynamic changes in this ratio. It is obvious that just when it starts to snap, the height reduces drastically, and at the snap, this ratio is undefined.

It is observed that the H/W ratio is less than 1, indicating that the cross-sectional geometry of TNTs deviates from a cylindrical profile and instead is a more flattened or elliptical shape. Notably, a general pattern emerged in which some TNTs exhibited a decrease in height following rupture events. The data from “Video V5,” marked with a light blue line, corresponds to Fig. 5. The reported height and H/W values correspond to local cross-sectional measurements rather than the full TNT length. For each TNT, a line profile (100 pixels in width) was drawn perpendicular to the TNT axis at a fixed spatial location, specifically at the site where rupture occurred. This fixed-position analysis avoids conflating spatial heterogeneity along the TNT with its temporal evolution. Although TNT width and phase profiles vary longitudinally, all measurements were consistently performed at the rupture site for each TNT. Therefore, the trends reported reflect relative temporal changes at a fixed spatial position rather than absolute geometric parameters averaged over the entire TNT length.

## Conclusion

This study presents the application of a partially spatially coherent QPM system for label-free, high-sensitivity characterization of tunneling nanotubes (TNTs) in migrating fish skin keratocytes. Using a five-frame phase-shifting approach, high-resolution phase maps were reconstructed, enabling quantitative assessment of TNT height and its temporal evolution. The measurements indicate that TNT heights typically range from approximately 100–900 nm. Analysis of the height-to-width ratio further suggests that TNTs exhibit a predominantly flattened rather than ideal cylindrical geometry.

Time-resolved imaging revealed heterogeneous structural behaviors: during the observation period, some TNTs maintained structural integrity throughout the entire recording, whereas others underwent rupture. Tracking the temporal height dynamics, particularly in thinner TNTs, demonstrated progressive variations preceding snapping events. These results highlight the mechanically dynamic nature of TNTs and underscore the importance of high-sensitivity, time-lapse phase imaging for resolving nanoscale structural changes.

Note that the primary objective of this work was to demonstrate the capability of partially spatially coherent QPM to quantify TNT nanoscale geometry and monitor its evolution over time. While descriptive comparisons between intact and ruptured TNTs were performed, the study does not aim to establish population-level statistical significance. Therefore, larger-scale biological investigations will be necessary to derive statistically robust conclusions regarding TNT stability and function. Nevertheless, the present work provides a proof-of-principle demonstration of QPM as a powerful tool for investigating TNT morphology and dynamics in live cells and offers a quantitative foundation for future studies on intercellular communication mechanisms in fish.

## Supplementary Information

Below is the link to the electronic supplementary material.

Supplementary Material 1

Supplementary Material 2

Supplementary Material 3

Supplementary Material 4

Supplementary Material 5

Supplementary Material 6

Supplementary Material 7

Supplementary Material 8

## Data Availability

All data generated or analysed during this study are included in this article and its supplementary information files. The raw datasets used and/or analysed during the current study are available from the corresponding author upon reasonable request.
