# Supplementary material for: Real-time snapping dynamics and nanoscale thickness profiling of salmon keratocyte tunneling nanotubes using partially coherent quantitative phase microscopy
Source: Sci Rep. 2026 Apr 17;16:17808. doi: 10.1038/s41598-026-46064-1 (PMC13247071; doi:10.1038/s41598-026-46064-1)
Supplement: Supplementary file 1 — Supplementary Material 1 [file 41598_2026_46064_MOESM1_ESM.docx]

**Supplementary file**

**Real-time snapping dynamics and nanoscale thickness profiling of salmon keratocyte tunneling nanotubes using quantitative phase microscopy**

Bilal M. Afzal^1^, Marie K. Mikkelborg^1^, Dhivya B. Thiyagarajan^1^, Deanna L. Wolfson^2^, Balpreet S. Ahluwalia^2^, Roy A. Dalmo^1,*^, and Azeem Ahmad^2, *^

^1^The Norwegian College of Fishery Science, UiT The Arctic University of Norway, 9037, Tromsø, Norway

^2^Department of Physics and Technology, UiT The Arctic University of Norway, 9037, Tromsø, Norway

Corresponding author: [*azeem.ahmad@uit.no](mailto:*azeem.ahmad@uit.no) and [roy.dalmo@uit.no](mailto:roy.dalmo@uit.no)

**Supplementary-I**

**Supplementary Fig. S1.** Changes over time in the height of 10 frames from a representative movie, including one frame captured immediately after a snapping event. (a_1_ – j_1_) Reconstructed phase map images, with a yellow box highlighting the specific ROI measured. (a_2_ – j_2_) show the measured area (yellow box) within the specific ROI, with measurements taken across 100 pixels perpendicular to the length to determine the GH. (a_3_ – j_3_) Line profiles measured from the ROIs illustrate the depletion in height over the 10 frames.

**Supplementary-II**

Video V1 represents the timelapse sample-free interferometric movie recorded at 33 frame/s to measure the temporal phase stability of the system.

Videos V2 – V7 represent different phase movies of fish cells with their TNTs.
